# Health problems and disability in long-term sickness absence: ICF coding of medical certificates

**DOI:** 10.1186/1471-2458-11-860

**Published:** 2011-11-11

**Authors:** Roland Morgell, Lars G Backlund, Britt Arrelöv, Lars-Erik Strender, Gunnar H Nilsson

**Affiliations:** 1 Center for Family and Community Medicine, Department of Neurobiology, Care Sciences and Society, Karolinska Institutet, Stockholm, Sweden

## Abstract

**Background:**

The purpose of this study was to test the feasibility of International Classification of Functioning, Disability and Health (ICF) and to explore the distribution, including gender differences, of health problems and disabilities as reflected in long-term sickness absence certificates.

**Methods:**

A total of 433 patients with long sick-listing periods, 267 women and 166 men, were included in the study. All certificates exceeding 28 days of sick-listing sent to the local office of the Swedish Social Insurance Administration of a municipality in the Stockholm area were collected during four weeks in 2004-2005. ICD-10 medical diagnosis codes in the certificates were retrieved and free text information on disabilities in body function, body structure or activity and participation were coded according to ICF short version.

**Results:**

In 89.8% of the certificates there were descriptions of disabilities that readily could be classified according to ICF. In a reliability test 123/131 (94%) items of randomly chosen free text information were identically classified by two of the authors. On average 2.4 disability categories (range 0-9) were found per patient; the most frequent were 'Sensation of pain' (35.1% of the patients), 'Emotional functions' (34.1%), 'Energy and drive functions' (22.4%), and 'Sleep functions' (16.9%). The dominating ICD-10 diagnostic groups were 'Mental and behavioural disorders' (34.4%) and 'Diseases of the musculoskeletal system and connective tissue' (32.8%). 'Reaction to severe stress and adjustment disorders' (14.7%), and 'Depressive episode' (11.5%) were the most frequent diagnostic codes. Disabilities in mental functions and activity/participation were more commonly described among women, while disabilities related to the musculoskeletal system were more frequent among men.

**Conclusions:**

Both ICD-10 diagnoses and ICF categories were dominated by mental and musculoskeletal health problems, but there seems to be gender differences, and ICF classification as a complement to ICD-10 could provide a better understanding of the consequences of diseases and how individual patients can cope with their health problems. ICF is feasible for secondary classifying of free text descriptions of disabilities stated in sick-leave certificates and seems to be useful as a complement to ICD-10 for sick-listing management and research.

## Background

Despite improvements in medical technology, longer life expectancy and better objective measures of health, claims for sickness benefits are high in Sweden and other Western countries, especially among women [1,2]. Musculoskeletal disorders and psychiatric problems are the most common causes of sickness absence, long-term work incapacity and retirement due to ill-health among both men and women. An increasing proportion of claims is due to conditions characterised by subjective complaints, often with little objective pathology or impairment [2]. The aetiology of sick-listing absence is multifactorial, comprising both medical and non-medical components [3], and the prevalence and duration of sickness absence cannot always be explained by medical reasons alone [4].

In Sweden as well as in other Western countries, medical certificates issued by physicians constitute the basis for decisions regarding sickness benefits and vocational rehabilitation. The processes of sick-listing and rehabilitation involve different professional categories with different training and functions, and communication between them occurs mainly in writing, on special forms. The right to sick-leave cash benefits for the individual patient is in Sweden determined by the employer for the first two weeks of absence, and thereafter by a case officer at a governmental agency, the Social Insurance Administration (SIA). Non-employed patients are reimbursed by the SIA for the entire period. In both cases a physician has to state on a medical certificate that the patient cannot work because of a disease or an injury. On this form the physician has to indicate the patient's main medical ICD-10 diagnosis code.

A description of the patient's functional problems and capabilities is also a crucial part of the certificate, and this is done by means of free text. The physicians' certificates have been found to have a substantial impact on the decisions on sick-listing benefits made by the SIA officers [5,6]. Earlier studies have found that physicians perceive sickness certification as problematic, and that sickness certificates are often of poor quality [7-13]. Work ability assessment is one of the most problematic aspects reported by physicians [13].

The International Statistical Classification of Diseases and Related Health Problems (ICD) is the standard diagnostic classification for epidemiological and health management purposes [14,15]. All national and local statistics on sickness absence morbidity are based on the coded main diagnosis. Little is known about co-morbidity or health problems in a wider sense in sickness absence. Sick-listing statistics based on ICD-10 have shown that for both men and women the two most common diagnostic groups in Sweden are musculoskeletal and psychiatric problems [16].

The International Classification of Functioning, Disability and Health (ICF) is a WHO classification intended to be a tool for health statistics and research, as well as for clinical use and health care planning. ICF provides a multi-dimensional model of health and health related domains [17]. ICF consists of four components: 1) Body Functions (BF) and Body Structures (BS), 2) Activities and Participation, 3) Environmental Factors, and 4) Personal Factors (not yet developed). Each component has 5-9 domains of functioning (BF and BS) or environmental factors (i.e. chapters). A disability is an umbrella term for impairments (BF and BS), activity limitations and participation restrictions that consist of up to 20 categories, which are the coding level. For BF and BS ICF provides a system for classification of data at four levels of detail and a system of qualifiers that may be used to classify the degree of disability (i.e. no, mild, moderate, severe or complete). The ICF short version is limited to the second level [18]. ICF is not based on the concepts of disease; it includes neither causes of disability nor prognostic evaluation. ICD-10 and ICF are complementary, and WHO encourages health professionals to use these two classifications together, even though they can be used separately. ICD-10 includes symptoms and health related problems in addition to diseases, so ICD-10 and ICF are partly overlapping.

There is a steady increase in the published research on clinical applications of ICF, not least the development of ICF-based assessment tools, and on theoretical issues since its introduction in 2001[19].

Functional status has been found to be a better indicator of health care needs than diagnosis [20]. However, knowledge about functional problems among sick-listed patients is scanty and ICF is yet not commonly used in health care statistics. A recent review article [21] found more than one hundred articles between 2001 and 2008 with attempts to link health related text-based information to ICF. To our knowledge, only one previous study has mapped information in sickness certificates to ICF: A recent Swedish study [22] found that sickness certificates seem to provide scarce information on functioning and that the descriptions mainly concerned body components.

The primary aim of this study was to test the feasibility and reliability of ICF for classifying and coding disabilities as reflected in sick-leave certificates. A secondary aim was to explore the distributions of health problems and disabilities (i.e. regarding body functions, body structures, activities and participation) that cause work incapacity among men and women.

## Methods

### Setting

Norrtälje is a local community including a small town and surrounding rural area in the northern part of Stockholm County. There is a population of about 53,000, half of whom are aged 20-64 years, and less than 10% are first generation immigrants. The community includes one local hospital, three psychiatric centres, two clinics for alcohol and drug problems, and five primary health care centres. Some employers provide their employees with occupational health care. Advanced specialist care is available at university hospitals located about 50 km from the community.

### Data collection

During three periods (4 weeks together) all sick-leave certificates (n = 676) that were sent to the SIA office in Norrtälje were collected. The first period comprised two weeks in July 2004, the second period was one week in February 2005, and the third period was one week in May 2005. The periods were selected to represent different parts of the year, thereby reducing the risk of seasonal variations.

In a first step, we copied 675 certificates (one copy was excluded because it could not be read due to poor copy quality) concerning 582 patients (256 men and 326 women) relating to 593 episodes of sickness leave. In a second step, we selected 435 episodes that were classified as long-term sickness absence (defined as more than 28 days from the day the patient reported being sick to the SIA office until the day the certificate was issued), and for each episode of sickness absence the information in the last issued certificate was retrieved. In a third step, only the last episode was taken into account when a patient had more than one long-term episode (two women had two long-term sickness episodes each), resulting in a study population of 433 patients (166 men and 267 women). For each patient background data (age, sex, date, type of medical facility and ICD-10 main diagnosis code) were recorded.

### ICF classification and coding

In a pilot study classification and coding of free-text information in sick-listing certificates was done according to the guidelines for using ICF short. The pilot study made it clear that the free text information in the certificates was not explicit enough to support classification of degree of disability; in most cases there was just a description of what kind of disability the patient suffered from. Also, there was very seldom any information on environmental factors that could be used for ICF coding.

All information except the main diagnoses and environmental factors were classified and coded by one of the authors (RM), who is a family physician with previous ICF training. Ambiguous information was disregarded, and information on normal findings was not classified. Neither was information that the patient cannot work because of his disease classified, as the issuing of the form in itself provides that information. The main medical ICD -10 diagnosis was not classified according to ICF also in those cases that would have been possible (e.g. ICD diagnosis hypertension could be classified by ICF as blood pressure functions). RM was not blinded to the ICD codes. The core sets that previously have been proposed for various conditions were not used; on the contrary were all explicit deviations coded regardless of diagnosis. After the main coding a test of the inter-rater reliability in ICF coding was performed.

### Statistical analysis

The data was analyzed using the STATA^® ^software programme. Pearson chi-square was used to test distribution of cross-classified nominal variables. A p-value of <0.05 was considered statistically significant.

### Ethical committee

Approval was obtained from The Regional Ethics Committee of Stockholm.

## Results

### Background data

A total of 433 (= N) patients were included, each of them with one corresponding certificate, 166 (38.3%) men (mean age 45.8 years, range 21.6-70.0) and 267 (61.7%) women (mean age 45.8 years, range 20.7-64.8). General practitioners at PHC centres issued 55.1% of the certificates, 22.6% were issued at a hospital, 10.2% were issued by a physician at one of the local psychiatric health centres, and 9.2% at an occupational health centre.

### Quality of certificates

The majority of the certificates were printed while some of them were written by hand; all of them were possible to read. There were big differences in information content. Ten percent of the certificates did not provide any information on disabilities besides a medical diagnosis, some certificates provided scarce information, and other certificates had long free text descriptions.

### Medical diagnoses

The main ICD-10 diagnosis was coded in 431 of the 433 certificates. In this paper diagnostic codes with a very low frequency (< 1%) are included at ICD-10 chapter of disorder level, but not presented as specific diagnostic codes. The most frequent ICD-disorders were 'Mental and behavioural disorders' (34.4% of the patients), and 'Diseases of the musculoskeletal system and connective tissue' (32.8% of the patients) (Table 1). Each of the other disorders accounted for not more than 6.7% of the main medical diagnoses. The most frequent single diagnostic codes were F43 'Reaction to severe stress, and adjustment disorders' (14.7%), F32 'Depressive episode' (11.5%), and M54 'Dorsalgia' (9%). Table 1 shows the frequencies at chapter level together with diagnostic codes with significant gender differences.

**Table 1:** Distribution of ICD-10 chapters among the 443 patients.

ICD- 10 chapter	Code	All N = 433	Men N = 166	Women N = 267	Gender difference, p-value
	Missing	2 (.5)	1 (.6)	1 (.4)	

V. Mental and behavioral disorders		149 (34.4)	46 (27.9)	103 (38.4)	.03*

	F 43 Reaction to severe stress and adjustment disorders	64 (14.7)	16 (9.7)	48 (17.9)	.02*

XIII. Diseases of the musculo-skeletal system and connective tissue		142 (32.8)	55 (33.3)	87 (32.5)	.85

	M 54 Dorsalgia	39 (9.0)	22 (13.3)	17 (6.3)	.01*

XIX Injury, poisoning, and certain other consequences of external causes		29 (6.7)	16 (9.7)	13 (4.9)	.05

XVIII Symptoms, signs and abnormal clinical and laboratory findings		21(4.8)	10 (6.1)	11 (4.1)	.36

X Diseases of the respiratory system		15 (3.5)	7 (4.2)	8 (3.0)	.49

IX Diseases of the circulatory system		14 (3.2)	7 (4.2)	7 (2.7)	.35

II Neoplasms		12 (2.8)	5 (3.0)	7 (2.7)	.77

VI Diseases of the nervous system		8 (1.8)	4 (2.4)	4 (1.5)	.49

XI Diseases of the digestive system		11 (2.5)	5 (3.0)	6 (2.2)	.76

XXI Factors influencing health status and contact with health services		8 (1.8)	1 (.6)	7 (2.6)	.16

Remaining chapters (I,III,IV, VII,VIII, XII,XIV, XV, XVII)		22 (5.1)	8 (4.8)	15 (5.5)	.86

Diagnoses with significant gender differences are shown.

* p < .05, Chi-square or Fisher's exact test.

### Feasibility of ICF coding

There were 412 (95.2%) patients who had certificates with at least one description of a disability that readily could be classified and coded according to ICF Short. For the remaining 21 (4.8%) certificates there was no explicit and unambiguous information on disabilities other than the information provided by the medical diagnoses themselves, or a general statement that the patient cannot work during the period of sick-listing.

### Reliability of ICF coding

A reliability test was performed, where two of the authors (LGB and RM) independently classified free text information in 50 randomly selected certificates. The result was that 123/131 (94%) items of free text information were identically classified according to ICF short version.

### ICF Components and domains

The most frequent component was 'Body function', which was described in 83.4% of the patients (Table 2). The corresponding figures were 83.1% for men and 83.5% for women (non-significant). 'Activity and participation' was described in 35.1% (32.5% of the men, and 36.3% of the women, non-significant) and 'Body structure' in 19.2% (23.5% of the men and 16.8% of the women, p = 0.07).

**Table 2:** Number (%) of patients with components and domains of functioning according to ICF.

ICF components and domains	All (N = 433)	Men (N = 166)	Women (N = 267)	Gender difference, p-value
*Body functions*	361 (83.4)	138 (83,1)	223 (83,5)	1

1. Mental functions	219 (50.6)	73 (44)	146 (54.7)	.03*

*Body structures*	83 (19.2)	39 (23.5)	44 (16.8)	.07

1. Structures of the nervous system	6 (1.4)	5 (3)	1 (0.4)	.03*

7. Structures related to Movements	61 (14.1)	32(19.3)	29 (10.9)	.01*

*Activities and participation*	152 (35.1)	54 (32.5)	98 (36.7)	.38

2. General tasks and demands	65 (15)	16 (9.6)	50 (18.7)	.01*

Domains with significant gender differences are shown.

* p < .05, Chi-square or Fisher's exact test.

On average, 1.7 domains of functioning were described per patient, ranging from 0 to 5. In 'Body function', the most frequently described domains were 'Mental functions' (50.6%), followed by 'Sensory functions and pain' (37.9%) (Table 2 showing domains with significant gender differences). In the 'Activity and participation' component, the most frequently described domain was 'Mobility' (17.8%).

### Categories

On average, 2.4 categories were described per patient, ranging from 0 to 9. The most frequent categories were 'Sensation of pain' (35.1%), 'Emotional functions' (34.1%) and 'Energy and drive functions' (22.4%) (Table 3 only categories with an occurrence of at least 5% are shown). As for gender differences, impairments in 'Emotional functions' and 'Handling of stress and other psychological demands' were more frequent among women, while impairments in 'Structure of trunk' were more common among men.

**Table 3:** Distribution of ICF categories among men and women and among patients with a mental or musculoskeletal ICD diagnosis.

ICF category	All (N= 433)	Men (N = 166)	Women (N = 267)	Gender difference,p-value	Mental (ICD) N = 149	Musculo-skeletal (ICD) N = 142	ICD **difference**, p-value
Sensation of pain (b180)	152 (35.1)	57 (34.3)	95 (35.6)	.79	19 (12.8)	95 (66.9)	.00*

Emotional functions (b152)	148 (34.1)	45 (27.1)	103 (38.6)	.01*	99 (66.4)	25 (17.6)	.00*

Energy and drive functions (b130)	97 (22.4)	30 (18.1)	67 (25.1)	.08	58 (38.9)	10 (7)	.00*

Sleep functions (b134)	73 (16.9)	23 (13.9)	50 (18.7)	.19	47 (31.8)	12 (8.5)	.00*

Handling of stress and other psychological demands (d240)	64 (14.8)	16 (9.6)	48 (18)	.02*	51 (34.5)	6 (4.2)	.00*

Attention functions (b140)	54 (12.5)	16 (9.6)	38 (14.2)	.16	43 (28.6)	2 (1.4)	.00*

Mobility of joint functions (b710)	50 (11.5)	24 (14.5)	26 (9.7)	.14	1 (.7)	36 (25,4)	.00*

Walking (d450)	27 (6.2)	10 (6)	17(6.4)	.89	1 (.7)	19 (13.4)	.00*

Structure of trunk (s760)	26 (6)	17 (10.2)	9 (3.4)	.00*	1 (.7)	22 (15.5)	.00*

Lifting and carrying objects (d430)	23 (5.3)	13 (7.8)	10 (3.7)	.07	0 (0)	16 (11.3)	.00*

Maintaining body position (d415)	22 (5.1)	9 (5.4)	13 (4.9)	.80	1 (.7)	18 (12.7)	.00*

Categories with a frequency of at least 5% are shown.

* p < .05, Chi-square or Fisher's exact test.

### Disability and disease correspondence

As expected, patients with an ICD-10 diagnosis indicating a mental or behavioural disorder, or a disease of the musculoskeletal system or connective tissue, in most cases also had some ICF category disability related to the same body system. For each of the most common ICF categories the differences in occurrence between the ICD chapters 'Diseases of the musculoskeletal system and connective tissue' and 'Mental and behavioural disorders' were highly significant. The differences between descriptions of disabilities in body function, body structure or activity and participation were however not restricted to patients with diagnoses in the same body system. For example, the most frequent impairment of body function 'Sensation of pain' was found in 66.9% of patients with 'Diseases of the musculoskeletal system and connective tissue' and in 12.8% of patients with 'Mental and behavioural disorders' (Table 3).

### Gender perspective

The majority of patients were women (62%). For both men and women mental and musculoskeletal ICD diagnoses were the most common categories. For women ICF impairments related to mental problems were more pronounced, for men descriptions of impairments related to the musculoskeletal system were relatively more common.

## Discussion

Our study emphasises the importance of previous findings that sickness and impaired work capacity are complex and multidimensional phenomena that are not captured solely by main diagnoses [3,4,20]. ICF is feasible for classifying information on disabilities in structures, functioning and activity and participation in sick-listing certificates. ICF classification thus seems to be a good complement to ICD-10 as it provides additional information concerning functioning and disabilities.

Our findings that a majority of patients have 'Mental and behavioural disorders' or 'Diseases of the musculoskeletal system and connective tissue' are consistent with previous data [16].

Our findings on component level revealed gender differences (e.g. that there were for men more frequent descriptions of 'Body structure' impairments). This may be due to different morbidity, but may also reflect physicians' attitudes and practices. It would be of interest to further analyse the distributions of ICF categories given the same ICD diagnoses from a gender perspective. However, in the present material the vast majority of ICD diagnoses contained less than 15 patients which makes it difficult to reveal differences. The most frequently described domain, 'Mental functions', was present for 50.6% of the patients which was higher than expected when relating to ICD-10 chapters, although there are no studies available for comparison of ICF figures. The most frequent single disabilities ('Sensation of pain', 'Emotional functions' and 'Energy and drive functions') were somewhat expected. The fairly high figures (1.7 and 2.4) for average number of domains and categories of disability underline the multifaceted nature of sickness absence mentioned above [3].

The overlapping between mental and physical diseases and disabilities found in this study, e.g. that 'Diseases of the musculoskeletal system and connective tissue' frequently had mental components, was somewhat greater than expected. These findings clearly show that a medical diagnosis alone does not provide enough information in the areas of sick-leave management and rehabilitation, and that the severity and impact of a disease can vary considerably. As mentioned above, such disease and disability overlap in sickness absence has received little attention, and to our knowledge there are no studies available for comparison (although the previously cited Swedish study [22] describes the overlap at the component level).

A general limitation is that we had no first-hand information on the sick-listed patients' health status, since our source of knowledge was information in medical certificates. As the aim of these certificates is to describe why the patient cannot work and is thus entitled to cash benefits, we believe that our figures are fairly accurate concerning directly related disease and disability. However, it is our experience that physicians describe only the disabilities they consider to be of relevance for sick-listing in a limited perspective, and our figures are consequently an underestimation of the total individual disability. Further, about 10% of the certificates had no information on disability, and this is a limitation, although it is considered minor. The main strength is the retrospective approach using records and certificates from everyday clinical practice, thereby excluding any recording bias.

The high prevalence of disabilities, the overlapping between mental and physical diseases and disabilites, and the high frequencies of multiple disabilities have implications for the management and rehabilitation of these patients. The findings emphasise the need to expand the traditional medical-legal one-diagnosis approach to include functional assessment and recognition of multiple disabilities in rehabilitation in long-term sickness absence, and ICF can be used to support this approach. However, assessing and describing patients' functioning and activities and their participation in everyday life is not part of the traditional training of physicians, and they regard such activities as problematic [7-9]. There is therefore a need for such training to be included in the medical education of physicians.

We found that the free-text descriptions in the certificates could in most cases be easily classified according to the ICF short version, and our results might be used as a basis for a check-list. ICF can also facilitate and provide basic concepts for communication between doctors, patients and the SIA, which is shown to be needed in rehabilitation in long-term sick-listing [5,6]. For a number of chronic conditions a selection of relevant (core set) ICF items has been proposed by international expert groups [23-28]. Such a core set has recently been developed in the Norwegian Functional Scale to help health professionals assess patients' functions in the sick-listing process [29]. ICF can also be useful, in combination with ICD-10, for epidemiological descriptions of health problems as well as in related research [30].

## Conclusions

For both men and women long-term sickness absence is dominated by mental and musculoskeletal diseases. The domains of impairment of the body functions were even more predominated by the mental and neuromuskuloskeletal system, and were more multifaceted. The ICF was feasible for describing impairments stated in sick-leave certificates, and seems useful, as a complement to ICD-10, for clinical practice and research concerning long-term sickness-absence, which has implications for medical education and the recognition of multiple impairments in long-term sickness absence rehabilitation.

## Competing interests

The authors declare that they have no competing interests.

## Authors' contributions

RM contributed to the design and coordination of the study, participated in the acquisition of data, analysed and classified the data, and wrote the manuscript. BA, LES, LGB and GN contributed to the design of the study, analysis of the data, and drafting of the manuscript. All authors read and approved the final manuscript.

## Pre-publication history

The pre-publication history for this paper can be accessed here:

http://www.biomedcentral.com/1471-2458/11/860/prepub
